# Gastric dysrhythmias in patients with early systemic sclerosis: a cross-sectional study

**DOI:** 10.1093/rap/rkae041

**Published:** 2024-03-12

**Authors:** Daniela Seelmann, María Paz Poblete, Silvana Saavedra, Ana María Madrid, Christian von Muhlenbrock, Camila Estay, Annelise Goecke

**Affiliations:** Rheumatology Section, Medicine Department, Hospital Clínico de la Universidad de Chile, Santiago, Chile; Rheumatology Section, Medicine Department, Hospital Clínico de la Universidad de Chile, Santiago, Chile; Rheumatology Section, Medicine Department, Hospital Clínico de la Fuerza Aérea de Chile, Santiago, Chile; Gastroenterology Section, Medicine Department, Hospital Clínico de la Universidad de Chile, Santiago, Chile; Gastroenterology Section, Medicine Department, Hospital Clínico de la Universidad de Chile, Santiago, Chile; Digestive Disease Center, Internal Medicine Department, Universidad de los Andes, Santiago, Chile; Gastroenterology Section, Medicine Department, Hospital Clínico de la Universidad de Chile, Santiago, Chile; Rheumatology Section, Medicine Department, Hospital Clínico de la Universidad de Chile, Santiago, Chile

**Keywords:** systemic sclerosis, nailfold capillaroscopy, gastrointestinal disorder, electrogastrography

## Abstract

**Objectives:**

Gastric involvement in patients with early systemic sclerosis (SSc) has not been previously investigated. We aim to evaluate the association of gastric dysrhythmias with gastrointestinal (GI) symptoms and nailfold video capillaroscopy (NVC).

**Methods:**

Cross-sectional study. Patients with early SSc, completed the UCLA GIT 2.0 questionnaire, performed an NVC, and a surface Electrogastrography (EGG). Descriptive statistics was used for demographic and clinical characteristics and Fisher and Kendall Tau tests were used for association analysis.

**Results:**

75 patients were screened, 30 patients were consecutively enrolled, 29 performed the EGG and 1 patient had a non-interpretable NVC. 29/30 were female with a mean age of 48.7 years (25–72). The mean disease duration from the first non-RP symptom was 22.6 +/-10.8 months and most of the patients had limited disease (76.6%). Total GIT 2.0 score symptoms were moderate-severe in 63% of the participants and 28/29 had an abnormal EGG. Bradygastria was the most common pattern present in 70% of the participants. NVC patterns: 17% early, 34% active, 28% scleroderma-like, 14% non-specific, and 2 patients had a normal NVC. There was no association between severe GI symptoms or NVC patterns and severely abnormal EGG, but the presence of bradygastria was associated with severe impairment in the social functioning area (p 0.018).

**Conclusions:**

Gastric dysmotility is common in early SSc and there is a lack of correlation between GI symptoms and NVC scleroderma patterns. EGG is a sensitive, cheap, and non-invasive exam, that may be an alternative to early diagnosis of GI involvement.

Key messages

- Systemic sclerosis commonly affects the gastrointestinal tract and dysmotility is the primary abnormality.
- Severe symptoms were not associated with more severe abnormalities in electrogastrography.
- Electrogastrography may be a tool to accomplish early diagnosis of GI involvement in patients with systemic sclerosis.

## Introduction

Systemic sclerosis (SSc) is a multi-systemic autoimmune disorder characterized by small vessel vasculopathy and fibrosis of the skin and internal organs [1]. The gastrointestinal (GI) system is affected in up to 90% of patients during the disease course and recent data suggest that severe GI involvement may be present in more than 15% of SSc patients by the fourth year of the disease [2–4]. SSc-GI involvement may significantly affect quality of life, and in severe cases, is associated with higher mortality [3, 5, 6].

The primary pathophysiological abnormality in GI involvement is dysmotility [7], which is thought to be secondary to an immune-mediated inflammatory process and progressive vascular abnormalities that contribute to fibrotic changes, poor microcirculation, and altered contractility [8, 9].

Any region of the GI tract may be involved with gastric compromise reported in 10–75% of the patients [10]. The main symptoms of gastric dysmotility are nausea, vomits, abdominal distention, early satiety, and difficult-to-treat gastroesophageal reflux (GER). However, the correlation between symptom severity and objective severe gastric abnormalities is conflicting [11, 12].

GI dysmotility may be assessed by different methods, including Electrogastrography (EGG) a non-invasive, non-radioactive, safe, and economical one. The EGG measures trans-cutaneous gastric myoelectrical activity, and when impaired it predicts delayed gastric emptying with an accuracy of about 85% [13, 14].

Several studies have demonstrated that capillaroscopic changes are associated with the progression of symptoms and signs of organ involvement in SSc; with a direct relationship between the late-phase scleroderma pattern and a more severe systemic involvement. This has been shown for pulmonary involvement, pulmonary hypertension, skin ulcers, and esophageal involvement [15–19].

It is important to determine whether gastric dysmotility, as a surrogate marker of gastric involvement, is present in early SSc before irreversible damage is established, and if so, evaluate if it correlates with the presence and severity of GI symptoms.

Therefore, the primary aim of this study is to evaluate the prevalence of gastric dysrhythmias and its association with the presence of GI symptoms in patients with early SSc. A secondary aim is to evaluate the association of gastric dysrhythmias with the different capillaroscopic patterns.

## Methods

### Study design and population

In this cross-sectional study patients who fulfilled the SSc ACR/EULAR 2013 classification criteria [20] and had early disease (defined as disease duration <3 years since the onset of the first non-Raynaud’s symptom attributable to SSc) were included. Patients with a history of diabetes mellitus, and pregnant women were excluded.

Patients from different medical centers in Chile were invited to participate between May and October 2022 and consecutively selected if they fulfilled the selection criteria. All patients signed an informed consent. Demographic variables, laboratory tests, past and present medications, and internal organ involvement studies including echocardiogram, chest CT, pulmonary function tests, upper digestive endoscopy, and esophageal manometry were registered if available. The study was approved by the Ethics Committee of the Clinical Hospital of the University of Chile.

### GI tract assessment

Patients completed the UCLA GIT 2.0 questionnaire to identify regular experienced GI symptoms [21]. This questionnaire evaluates 7 areas (reflux, distention/bloating, fecal soilage, diarrhea, social functioning, emotional well-being, and constipation) and the final score goes from 0 (no symptoms) to 3 (severe symptoms). The severity was assessed using the scale provided by Khanna *et al.* [22]

The gastric myoelectrical activity was assessed within an interval no longer than 30 days from the UCLA GIT 2.0 questionnaire, with a surface EGG (Medtronic-Synectics, Medical ^®^, Stockholm, Sweden). All EGG was done in the same place, in a standardized way by the same technician (AS), with the same equipment, and informed by a gastroenterologist blinded to patients' identity. Two bipolar electrodes were placed, one halfway between the navel and the xiphoid process and the other 5 cm/45 degrees to the left of the first one. A reference electrode was also placed. Prokinetics and macrolides had to be discontinued at least 15 days before the exam and a 12-h fasting before the exam was required. The register included a 60-minute basal and postprandial period after a standardized meal of 350 kcal.

Dominant frequencies and percentual distribution in pre- and post-prandial periods were registered as well as the Power Ratio (ratio between pre and postprandial amplitude). Analysis was made with the Fourier computerized method. A normal study should have more than 70% of the electrical activity with frequencies between 2.4 and 3.7 cycles per minute (cpm) and a Power Ratio (PR) between 1 and 10 microvolts. Dysrhythmias were informed as continuous or predominantly pre or postprandial, with a predominance of bradygastria, tachygastria, or mixed patterns. The results of SSc patients were compared with EGG previously done in our same institution and by the same team, in subjects with functional dyspepsia and healthy asymptomatic controls [23].

As there is no standardized way of classifying the severity of the abnormalities in EGG, we arbitrarily grade it in 2 different ways, to facilitate statistical analysis and generate clinically relevant data. First, we defined severe compromise as the presence of continuous bradygastria, a criterion that has been used in previous studies [12]. As a second severity criterion, we applied a score designed by one of the authors (A.M.) before data analysis. This score conferred 1 point for abnormal dominant frequency (pre- and post-prandial) and percentual frequency distribution (pre- and post-prandial); and 2 points for an abnormal PR. Final scores varied between 0 and 10, being mild (0–3), moderate (4–6), or severe (7–10) compromise defined arbitrarily for the study. This score has not been validated yet.

### Nailfold videocapillaroscopy (NVC)

The procedure was performed by two operators (M.P, D.S) after 15 min of rest in a room with a constant temperature of 20–22°C. Patients were asked to abstain from manicures at least 2 weeks before the exam. Optilia Video Capillaroscope was used and 2 pictures of each finger from the 2nd to the 5th of both hands were taken with a magnification of 200x. Images were analyzed by a blinded expert (S.S) using the Optipix Capillaroscopy Software. The following NVC parameters were evaluated on a semiquantitative scale: capillary density, hemorrhages, enlarged and giant capillaries, avascular areas, and organization of capillary architecture [24]. Scleroderma patterns (early/active/late), scleroderma-like patterns, and nonspecific patterns were also described [25].

### Statistical analysis

A sample size of 28 patients was calculated for an estimated 25–50% prevalence of gastric dysmotility, and a 5% precision in the assessment of the association between EGG abnormalities and GI symptoms [12]. Data was analyzed through Windows SPSS 22.0. Descriptive statistics were used to summarize baseline demographic and clinical characteristics. Continuous variables were presented with mean ± standard deviation (SD) when there was a normal distribution and as median and interquartile range when not. Categorical variables were presented with frequencies and percentages. For the main association analysis, Fisher and Kendall Tau tests were used. Statistical significance was defined with *p* < 0.05. Exploratory analysis using Spearman correlation was conducted to assess if there was a linear correlation between the UCLA GIT 2.0 score and the EGG severity score.

## Results

We screened 75 patients, of which 30 fulfilled the selection criteria signed the informed consent, and were enrolled. One participant did not have the EGG done and another patient had a non-interpretable NVC. Patients' characteristics are represented in Table 1. Most of the patients were female (29/30) with a mean age of 48.7 years (range 25–72). The mean disease duration from the first non-RP symptom was 22.6 +/-10.8 months and most of the patients had limited disease (76.6%).

**Table 1.** Demographic and baseline characteristics of study subjects

Demographic and Clinical Features	N = 30
Female, % (n)	96.6 (29)
Age, mean (range)	48.7 (25-72)
Limited SSc, % (n)	76.6 (23)
Diffuse Ssc, % (n)	23.3 (7)
Disease duration in months, mean (SD)	22.6 (10.8)
DMARD, % (n)	53.3 (16)
Methotrexate % (n)	23.3 (7)
Mycophenolate, % (n)	26.6 (8)
Rituximab, % (n)	3.3 (1)
Glucocorticoids, % (n)	43.3 (13)
Serology^a^	+ (N of patients with exam available)
ANA	28 (28)
Anti centromere	14 (28)
Scl-70	6 (24)
RNA pol III	1 (6)
PM-Scl 100	1 (6)
Organ involvement^b^	
Interstitial Lung Disease, % (n/N)	24 (7/29)
Abnormal Echocardiogram, % (n/N)	40 (10/25)
Erosive esophagitis, % (n/N)	73 (11/15)
Digital ulcers, % (n)	23.3 (7/30)
Joint disease, % (n)	50 (15/30)

Interstitial Lung Disease was evaluated by HRCT and PFT.

Abnormal echocardiogram referred to the presence of pulmonary hypertension (2 patients), pericardial effusion (2 patients), valvular insufficiency (1 patient), chamber dilation (3 patients), left ventricular hypertrophy (1 patient) or diastolic dysfunction (1 patient).

Erosive esophagitis was evaluated by upper endoscopy.

Digital ulcers and joint disease: Reported by the patients.

a Serology: ANA was measured by indirect immunofluorescence (IFI); Scl 70, RNA pol III, and PM-Scl were measured by ELISA.

b Organ involvement study information was not available in all patients; N represents the number of patients in which the information was available.

DMARD: Disease-Modifying Antirheumatic Drugs; ANA: Antinuclear antibodies; Scl 70: Antitopoisomerasa-1 antibodies; HCRT: High-resolution computed tomography; PFT: Pulmonary Function Tests.

Not all patients had an evaluation of internal organ involvement available. Interstitial lung disease was present in 24%, abnormal echocardiogram in 40%, and erosive esophagitis in 73% of the patients who had been studied for organ involvement. Digital ulcers and joint disease as reported by the patient were present in 23% and 50% respectively (Table 1). Antinuclear antibodies (ANA) were available and positive in 28 patients. Half of the patients were under immunosuppressive treatment, most of them receiving mycophenolate mofetil (MMF) or methotrexate (MTX). More detailed information can be found in Table 1.

###  

#### UCLA questionnaire results

The presence and severity of GI symptoms according to the UCLA GIT 2.0 questionnaire are shown in Table 2. Moderate to severe GI symptoms were present in 63% of the patients. Reflux and bloating/distention were moderate to severe in 53.3% and 56.7% respectively. Emotional well-being and social functioning were moderately to severely impaired in 56.7% of the patients.

**Table 2.** UCLA 2.0 GIT questionnaire scores [22]

Item	Normal—Mild n (%)	Moderate n (%)	Severe n (%)
Total GIT questionnaire	11 (36.7)	12 (40)	7 (23.3)
Reflux	14 (46.7)	9 (30)	7 (23.3)
Distention/Bloating	13 (43.3)	4 (13.4)	13 (43.3)
Diarrhea	16 (53.3)	11 (36.7)	3 (10)
Constipation	13 (43.3)	9 (30)	8 (26.7)
Faecal Soilage	25 (83.3)	3 (10)	2 (6.7)
Emotional Well-Being	13 (43.3)	6 (20)	11 (36.7)
Social Functioning	13 (43.3)	14 (46.7)	3 (10)

#### EGG results

**Figure 1.**
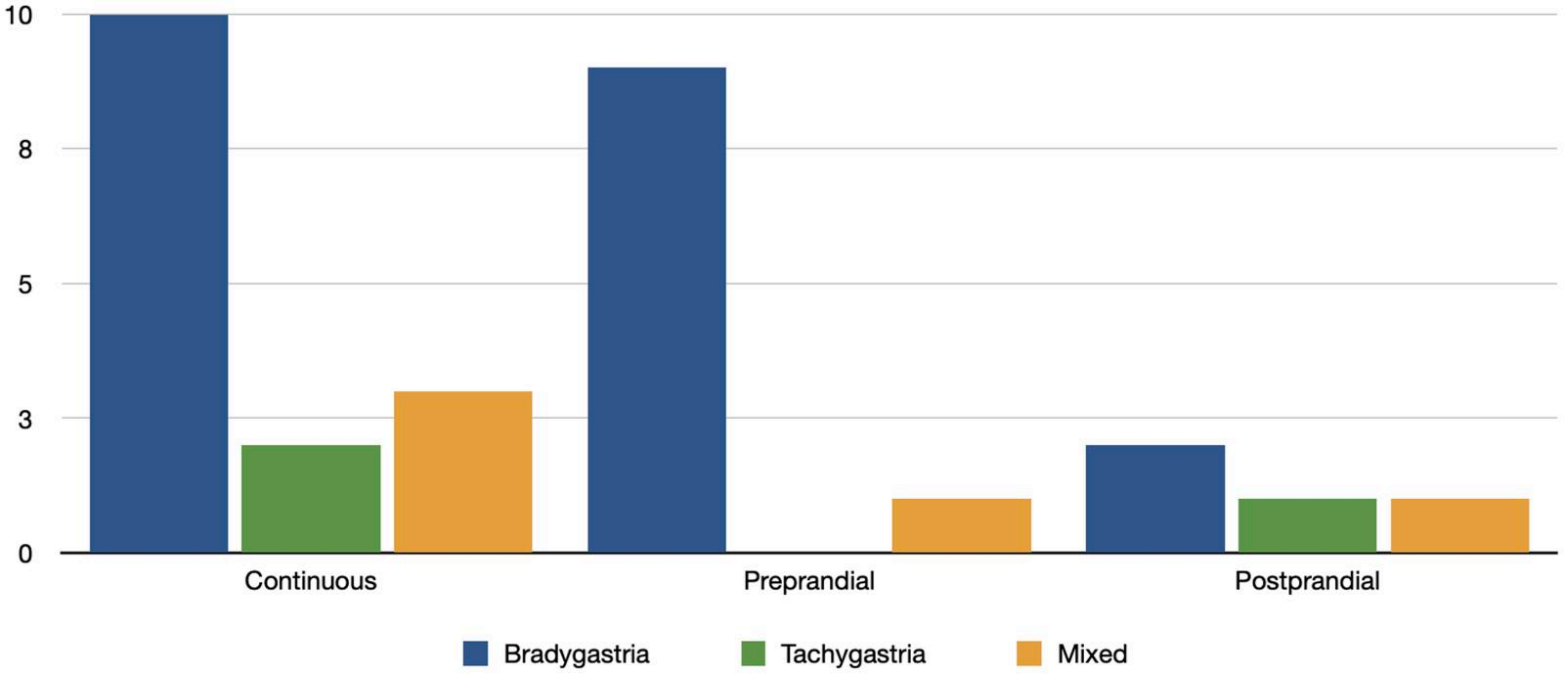
Types of gastric dysrhythmias. Bradygastria: Dominant electrical activity with frequencies between 0 and 2.4 cycles per minute (cpm). Tachygastria: Dominant electrical activity with frequencies between 3.8 and 10 cpm. Mixed pattern: Dominant electrical activity with bradygastria and tachygastria. Continuous dysrhythmia: Present in both preprandial and post-prandial periods. Please refer to the methods section for more detailed information

Before the exam, 2 patients had to discontinue domperidone, 1 patient prucalopride and there were no patients receiving opioids. Almost all patients (28/29) had an abnormal EGG. Eleven patients had preprandial dysrhythmia, 4 patients had postprandial dysrhythmia, and 12 patients had continuous dysrhythmia. In all of them, bradygastria was the predominant pattern. The Power Ratio was abnormal in 9/29 patients (decreased in 6, increased in 3). The different types of dysrhythmias in patients with SSc are represented in Fig. 1 and the comparison with the historic cohort of controls and patients with functional dyspepsia is in Table 3.

**Table 3.** Comparative prevalence and type of dysrhythmias [23]

Patients	Number	Age (Range)	Female (%)	Normal EGG (%)	Preprandial dysrhythmias (%)	Posprandial dysrhythmias (%)	Continuous dysrhythmias (%)
Control	10	36 (23-50)	5 (50)	9 (90)	1 (10)	0 (0)	0 (0)
SSc	29	48 (25-72)	28 (96.5)	1 (3.4)	10 (34.4)	4 (13.7)	15 (51.7)
Functional dyspepsia	57	45 (19-20)	44 (77)	22 (39)	16 (28)	10 (17.5)	9 (15.5)

Bradygastria which was present in 70% of the patients, was continuous in 50% of the patients, preprandial in 45%, and postprandial in 5%. Tachygastria was found in 10%, mixed pattern in 17%, and 1 patient had a normal exam (Fig. 1). According to the designed EGG severity score, 11 patients (37.9%) had a mild compromise, 11 (37.9%) moderate, 5 severe (17.24%) and only 1 patient had a normal score.

#### NVC results

NVC was done in the 30 participants, but it was uninterpretable in one of them because of poor visualization secondary to oedema. Semiquantitative analysis showed diminished capillary density in 15, giant capillaries in 21, and avascular areas in one patient.

Five patients (17%) had an early pattern, 10 had an active pattern (34%), 8 had a scleroderma-like pattern (28%),4 had a non-specific pattern (14%), and 2 patients had a normal NVC. No patient had a late scleroderma pattern.

#### Association analysis

We evaluated if there was an association between GI symptoms and EGG abnormalities. Our analyses showed that there was no association between severe GI symptoms and severely abnormal EGG defined as the presence of continuous bradygastria or a severe EGG score, as defined in the methods section. We specifically looked for an association between severe gastroparesis symptoms (reflux and/or distension/bloating) with severe EGG abnormalities, but it was not found.

35% of the patients with moderate to severe EGG abnormalities and 50% of the patients with continuous bradygastria were asymptomatic or had mild symptoms. An exploratory analysis using the Spearman correlation test did not show a linear correlation between ULCA GIT 2.0 scores and EGG severity scores. However, the presence of bradygastria was associated with severe impairment in the social functioning area (p 0.018). No association was found between EGG abnormalities and NVC patterns. Neither was an association between gastric dysmotility and SSc phenotype, specific autoantibodies, or internal organ involvement.

## Discussion

This is the first study that assesses gastric dysmotility and its association with GI symptoms and NVC scleroderma patterns in patients with early SSc. Our results show that patients with early SSc, regardless of scleroderma subtype, had a high prevalence (96%) of gastric dysmotility. This is higher than the previous reports with a similar number of participants where gastric myoelectric activity was abnormal in approximately 80% of SSc patients [12]. The most common EGG abnormality was bradygastria, especially continuous and preprandial bradygastria, which is concordant with previous descriptions for SSc patients. This predominance is thought to be secondary to a compensatory response in the postprandial period, which is lost in later stages, and not present in other conditions that also have altered myoelectrical activity [12, 14].

Despite not having a control group, we compared our results with a historic registry of one of the authors (AM). As shown in Table 3, healthy and asymptomatic patients usually have normal EGG, but patients with functional dyspepsia, have a high prevalence of EGG abnormalities, mostly preprandial, that have been associated with delayed gastric emptying in some studies [23].

Regarding symptoms, 63.3% of the subjects in our study had moderate-severe symptoms at baseline which is higher than what was reported previously (28%) [26]. However, the difference could be explained because the patient cohort studied by van Leeuwen et al. had a significantly shorter duration of the disease (median 5.9 months). Besides, ethnicity and environmental factors that may influence these results cannot be discarded. Severe EGG abnormalities were not associated with severe GI symptoms as assessed by the UCLA GIT 2.0 questionnaire. A correlation between higher scores in the UCLA GIT 2.0 questionnaire and the EGG severity score was not found. This goes in line with previous studies where there was also a lack of correlation between gastric dysmotility and GI symptoms in SSc patients [11, 27–29].

Considering this lack of correlation, it is important to highlight that 50% of patients with continuous bradygastria and 35% of the patients with a moderate-severe EGG score were asymptomatic or had mild symptoms.

We did find an association between the presence of bradygastria and severely altered social functioning area which is important to acknowledge. Altered social functioning area refers to altered social life secondary to GI symptoms, reflecting that severe symptoms negatively affect social life in patients with SSc.

Gut-brain interaction has been a topic of great interest over the past years, and the association between gut microbiota, neurotransmitters, and metabolites with different disorders such as depression, anxiety, and pain disorders, among others has already been demonstrated [30]. This gut-brain interaction could explain in part the association between bradygastria and altered social functioning, even though we did not find an association with the subitem of emotional well-being.

As mentioned before, capillaroscopic changes are related to the progression of symptoms and signs of organ involvement in SSc. Studies on NVC and GI involvement are scarce, and most of them focus on esophageal compromise. In our study, no association was found between NVC scleroderma patterns and gastric dysmotility or GI symptoms severity. Notably, we had a high percentage of scleroderma-like and nonspecific patterns (28% and 14% respectively). The reasons for the lack of correlation are not clear, maybe it is because our patients had early disease or was not captured because the sample was not big enough, or even could be a possible modulating effect of immunosuppressive therapies, which were used in half of the patients.

Our study has some limitations. We didńt have a control group, but as mentioned before we did compare with a historic control which showed that healthy and asymptomatic patients had normal EGG in 90% of the cases [23]. The cutoff of 3 years from the first non-Raynaud symptom may carry some limitations, first, it is data obtained from patients and may be subject to recall bias. It also may misclassify patients as having early systemic sclerosis, especially in patients with limited disease. However, none of the patients had a late scleroderma pattern. Another limitation is that symptom questionnaires have a subjective component that may have overestimated the severity symptoms score in our study. Regarding EGG we defined that it was more severe to have continuous bradygastria or a higher EGG score for analysis purposes, but even though the first criteria has been previously used in other studies [12], the second has not yet been validated. Abnormal EGG results should have been verified with a gold standard test as the scintigraphy, even though it is important to consider that some patients with symptoms of gastroparesis may have normal gastric emptying tests, and EGG may be altered earlier [28]. Lastly, it would have been useful to have concomitant esophageal manometry to assess if there is a positive correlation with gastric dysmotility and thus be able to optimize diagnostic procedures.

An important question that arises from our study is how to proceed with patients with subclinical disease, meaning patients with an abnormal EGG with none or mild symptoms. Most GI specialists would start treatment when symptoms develop, but it is known that this kind of dysmotility medication has better responses when the muscle isńt atrophied or fibrotic. Furthermore, dysmotility agents do not affect disease progression [31, 32].

There is limited and conflicting data regarding the use of immunosuppression or immunomodulation for the prevention or treatment of GI disease. Preclinical data and small observational studies suggest that immunomodulation with IVIG may benefit a subset of SSc patients with GI involvement, especially those with pseudo-obstruction [33, 34].

In a recent study by Richard et al., 762 subjects with early disease and without severe GI disease at baseline who received different immunosuppressive drugs were followed for 4 years. They found that the risk of severe GI disease (malabsorption, hyperalimentation, pseudoobstruction, and/or ≥10% weight loss in association with the use of antibiotics for bacterial overgrowth or esophageal stricture) was not modified by the exposure to immunosuppression [35]. The study is limited by the fact that only patients with severe GI disease at baseline were excluded, and it is not clear the baseline GI status of the other patients. Additionally, the exposure to immunosuppressive treatment was collected as nominal data, so there was no information about treatment duration or doses.

As a counterpart, to emphasize that more studies are needed Stamm et al., in an observational study that included 209 patients, showed that SSc patients who received immunosuppressive treatment (71) had lower UCLA GIT 2.0 scores than the ones without treatment at follow-up (12 ± 3 months) [36].

## Conclusion

Our study shows that gastric dysmotility is common in early SSc regardless of the phenotype and that there is a lack of correlation between GI symptoms and NVC scleroderma patterns. However, the presence of bradygastria was associated with severe impairment in the social functioning area of the UCLA GIT 2.0 questionnaire. We could consider EGG is a sensitive, cheap, and non-invasive exam, that may be a useful tool for early diagnosis of GI dysmotility or subclinical disease. It has been already stated that “there is an urgent need to study mild to moderate GIT disease and to identify preventive measures before it progresses to severe disease” [35]. Future studies should address whether asymptomatic EGG abnormalities are relevant and/or progressive, determine whether dysmotility in EGG is associated with dysmotility in other regions of the GI tract, and assess if treatment of asymptomatic abnormalities could lead to better outcomes or longer symptom-free periods. Finally, randomized clinical trials are needed to determine whether immunosuppressive treatment prevents the progression of GI disease.

## Data Availability

The data underlying this article will be shared on reasonable request to the corresponding author.
